# Progress in Diabetes

**Published:** 1902-03-29

**Authors:** 


### Progress in Diabetes.

At the Cheltenham meeting of the British Medical Association the causes leading to the production of glycosuria were tabulated by Pavy,[1] who groups the origin of sugar in the urine under four heads, viz.:—

1. Unassimilated carbohydrate.
2. Stored glycogen abnormally passing into sugar which thereafter finds its way into the urine.
3. Proteid matter—by an abnormal breaking-down process the sugar which has been linked to the proteid molecule becomes separated again and eliminated in the urine.
4. Its possible production from an abnormal condition of fat metabolism.

With regard to the first, carbohydrate assimilation has its limit; this is more readily overstepped by large ingestion of sugar in the normal body, than by that of starch. The morning urine two or three hours after the first meal may contain sugar when it is absent at other times of the day. Small quantities of sugar injected into the veins produce glycosuria, but no material effect upon the blood or urine within ordinary limits follows the injection of carbohydrate food. Carbohydrates, when in excess of requirements, may be converted into fat. This probably takes place in the villi and liver, and they are also probably built up, in the villi, into the compound proteid molecules produced during digestion. If they miss being converted into fat or incorporated into proteid they pass on to the liver and become glycogen: this is only supplementary to the assimilation elsewhere. Besides, in the villi protoplasm may continue to assimilate carbohydrate in the circulation provided enzymic action can take place. The kidneys have no share in the metabolism, only allowing the sugar to filter through. When cane sugar, lactose, or maltose are injected directly into the veins they are thrown out by the kidneys as useless, since they have met no enzymic action. Dextrose, lævulose, and galactose, on the other hand, are susceptible to assimilation.

It is thought that glycogen exists partly in loose combination with proteid material, viz., the nitrogenous nucleus, and that this can be oxidised off the protoplasm "ring" as a fatty "side chain" can be oxidised off a benzine ring leaving the latter intact. This may take place in muscles as well as in the liver.

Glycogen is converted into sugar after death. Bernard's puncture of the floor of the fourth ventricle may cause this transformation, as it fails when no glycogen is present. The probable explanation being that it produces vasomotor paralysis, allowing imperfectly de-arterialised blood to reach the liver by the portal vein: and this same occurrence may be the cause also of the villi being unable to assimilate the carbohydrate, and so leading to an excess of sugar finding its way into the portal vein. This is probably the explanation also of glycosuria following operations involving the sympathetic nerve.

The theory with regard to the production of sugar by means of the disruption of the proteid molecule, explains why the nitrogenous excretion varies with the sugar excretion. It also throws light upon those forms of diabetes depending upon interference with the pancreas or the ingestion of phloridzin, though in the latter the kidney undoubtedly plays some unknown part.

Arguing from the analogy of the vegetable kingdom, fat may be an antecedent of sugar, but opposed to this view is the fact that a fatty diet is usually beneficient in diabetic patients.

A rather different grouping of the origins of glycosuria is arranged by Professor Thompson.[2] He makes three classes.

(1) Excessive sugar in the blood due to over consumption or deficient oxidation (asphyxia, carbon-monoxide poisoning, chloroform, etc.).

(2) Renal glycosuria (phloridzin).

(3) Glycosuria due to a damaged glycogenic function of the liver (Bernard's puncture and various poisonings).

With regard to phloridzin, he holds that it either acts by rendering the kidney more sensitive to sugar in the blood, or else sugar is split off from it itself: yet if this were the sole cause, it would be expected that after the administration of the drug, if the ureters were tied, sugar would accumulate in the blood, but this does not happen. Phloridzin diabetes occurring during a purely flesh diet shows a constant relation between sugar and nitrogen excreted, a fact indicating a proteid source for the sugar. To explain the diabetes caused by complete removal of the pancreas, he puts forward the theory that the digestion of proteids being upset, poisonous substances are formed which throw the liver metabolism out of gear; and in support of this he states that:—

(1) In pancreatic diabetes a large excretion of aromatic sulphates occurs, indicating a perverted condition of proteid digestion.

(2) In cholera glycosuria often appears when urine excretion becomes re-established.

(3) The filtrate of a leucin-free artificial pancreatic digestion causes glycosuria when injected into animals. The same condition has been caused by the dialysate from the intestinal contents of diabetics.

(4) Kossel and Kutscher's "Arginine," a crystalline end-product of pancreatic proteolysis when given with food to one of two dogs produced glycosuria to the extent of over 1 per cent. of sugar: though using a smaller dose the second experiment failed.

(5) The condition takes some time to develop after the removal of the organ, and a much longer time to attain the maximum.

(6) Some cases of this affection are made much worse by giving chopped-up pancreas with the food.

Vaughan Harley[3] considers that under certain circumstances sugar appears in the urine simply because it has not been used up in the body metabolism. A patient of his was cured by simply attending to diet and regulating the bowels. So long as sugar did not increase in the urine he increases the carbohydrates in the diet.

A crystallisable substance has been isolated from the urines of diabetics and pneumonics by Lépine and Boulud,[4] which has the power of hindering sugar destruction. When this substance was mixed with normal defibrinated dog's blood and left for an hour at 38°–39° C. the blood was found to have lost very little of its reducing power, and very little of its fermentable sugar, while an unmixed specimen showed marked diminution in reduction power and almost complete disappearance of fermentable sugar. This substance is destroyed in its passage through the vessels of the living pancreas. And so in addition to its other functions the pancreas has an important antitoxic action.

The relation of the islands of Langerhaus in the pancreas to the production of diabetes mellitus has been before suspected by Opie;[5] he now records a case where this relation seems to be demonstrated. A coloured woman dying of acute tuberculosis had 4 to 5·4 per cent. of sugar in her urine. Post-mortem the brain, etc., were found normal, and the pancreas itself was very little involved by any degenerative change, but the islands of Langerhaus were found in advanced hyaline degeneration. He concludes that since pancreatic duct obstruction does not cause diabetes, though the secreting part of the gland undergoes great alteration, the islands being spared, the inference is justified with the evidence of this case before us, that these latter structures have some influence on carbohydrate metabolism.

Mossé[6] claims that certain of the patients suffering from diabetes, especially the arthritic form, are considerably benefited by the ingestion of potatoes in fairly large quantity instead of bread. He holds that glycosuria, polyuria and thirst, are diminished with marked improvement of the general health, healing of surgical wounds, etc. He states that many cases of arthritic diabetes can take a quantity of potato equivalent to 3,000 grains of starch without eliminating more than 500 to 600

grains of sugar, while the fæces are practically devoid of unassimilated starch. He holds that potatoes have a curative action owing to their contained alkaline (chiefly potassium) salts. He recommends their cautious administration and their result on the urine watched by frequent analysis.

It is now becoming generally recognised that diabetes patients behave very differently under the same diet, and Williamson[7] protests against the treatment of all cases alike, irrespective of cause, by very rigid diet and large doses of opium and codeia. The onset of complications, such as phthisis or granular kidney, will occasionally be attended by a diminution in the sugar excretion, and thus may wrongly be attributed to success in treatment. The amount of calories in the ingested food should be compared with those of the excreta, and the result should leave 2,300 calories, the amount required in health. Sudden changes in diet may predispose to coma in an advanced case; the urine should be carefully tested before dieting, and where this gives the perchloride of iron test great caution should be employed. Diet should be only strict enough to arrest sugar excretion. As soon as this is accomplished a more liberal dietary should be given. In dieting care should be exercised to see that the patient's weight does not decrease. In those cases giving the perchloride of iron reaction, albumen should be decreased and carbohydrates increased in the form of white bread and milk. He has prepared an artificial milk without sugar by dissolving out the sugar from cream with water, and mixing this cream in water with salt, white of egg, and saccharine.

For drugs he recommends opium, morphia, codeia, heroin hydrochloride, and sodium salicylate. He has found the latter markedly diminish sugar excretion, and also the same with aspirin. Bismuth salicylate induces constipation, but salol is useful. When coma is threatened or commencing he has found large doses of sodium bicarbonate of great use, 3 grains in 24 hours; and in three instances coma has been delayed for some weeks. Ensuing coma is indicated by rapidly-increasing weakness and loss of flesh, rapid pulse, and sudden appearance of a very large number of granular or hyaline casts in the urine, nausea, epigastric pain, air-hunger, and drowsiness.

Several writers testify to the value of the high frequency currents in the treatment of diabetes. Marshall[8] states that sugar excretion has been reduced almost to vanishing point, weight has increased, and general health completely restored. Dodd,[9] quoting Apostoli and Berlioz, states that sugar has been noticed to disappear almost entirely, and where this has not been so general improvement has occurred, insomuch that for the general improvement alone it is to be recommended. Another writer[10] has seen four cases get quite well, and one inveterate case improve markedly, but relapse after an interval of a few months. Manders[11] holds that in using the currents the cause of the glycosuria should be taken into consideration and the electrodes placed accordingly. Thus, in cases of cerebro-spinal origin (floor of fourth ventricle or cervical and upper dorsal regions) a small electrode should be placed close to the occiput and the current used weak at first and gradually increased as tolerated, the duration being 10 minutes. In cases of pancreatic origin the electrode should be large and situated over the head of the pancreas, the full current being used from the beginning for 15 minutes' duration. The temperature is the best guide as to the interval between the applications and the duration of treatment.

A new method of rapidly estimating the percentage of sugar in urine has been brought forward by Barker Smith.[12] It is an adaptation of his estimation of urea by means of the heat evolved when chlorinated soda is mixed with urine in the proportion of 5 to 1. He substitutes 4 cubic cent. of pot. permanganate solution (3 to 4 per cent.) with 1 cubic cent. of dilute sulphuric acid for the chlorinated soda, and reads off the percentage of sugar by multiplying the rise in temperature by 0·3. Thus if 1 cub. cent. of urine affords 10° F., the conclusion is that urine contains $10 \times 0{\cdot}3$ or 3 per cent. glucose. It must be borne in mind, however, that salicylic and carbolic acids and alcohol afford large amounts of heat during oxidation.

1 Brit. Med. Jour., Oct. 12. 2 Brit. Med. Jour., Oct. 12. 3 Brit. Med. Jour., Oct. 12. 4 Brit. Med. Jour., Feb. 1. 5 Amer. Jour. Med. Sci., September. 6 Brit. Med. Jour., Jan. 18. 7 Med. Chron. August. 8 Lancet, Oct. 12. 9 Lancet, Oct. 12. 10 Lancet, Oct. 12. 11 Lancet, Oct. 19. 12 Brit. Med. Jour., Nov. 23.

